# Health-Related Quality of Life among Artisanal Fisherwomen/Shellfish Gatherers: Lower than the General Population

**DOI:** 10.3390/ijerph13050466

**Published:** 2016-05-05

**Authors:** Juliana dos Santos Müller, Ila Rocha Falcão, Maria Carolina Barreto Moreira Couto, Wendel da Silva Viana, Ivone Batista Alves, Denise Nunes Viola, Courtney Georgette Woods, Rita Franco Rêgo

**Affiliations:** 1 Department of Technology in Health and Biology, Federal Institute of Education, Science and Technology of Bahia, Salvador 40301-015, Brazil; 2 Post-Graduate Program in Health, Environment, and Work, School of Medicine, Federal University of Bahia, Salvador 40026-010, Brazil; falcao.ila@gmail.com (I.R.F.); carolinabmcouto@gmail.com (M.C.B.M.C.); wendel_hp20@hotmail.com (W.S.d.V.); vonyssa@bol.com.br (I.B.A.); ritarego@ufba.br (R.F.R.); 3 Departmentof Statistics, Institute of Mathematics, Federal University of Bahia, Salvador 40170-115, Brazil; deniseviola@gmail.com; 4 Department of Environmental Sciences and Engineering, University of North Carolina at Chapel Hill, Chapel Hill, NC 27599-7431, USA; courtney.woods@unc.edu

**Keywords:** health-related quality of life (HRQOL), artisanal fisherwomen, SF-36, small-scale fishermen, chronic diseases, musculoskeletal disorders

## Abstract

Quality of life is an indicator of how well one perceives that he/she is functioning physically and mentally. The aim of this paper is to determine the health-related quality of life (HRQOL) of artisanal fisherwomen/shellfish gatherers from the Saubara municipality in Bahia, Brazil in comparison to the general population. A structured questionnaire was administered to a sample of 209 artisanal fisherwomen selected at random. The HRQOL questionnaire, known as the 36-Item Short-Form Health Survey version 1 (SF-36v01), was also used, having been translated and verified cross-culturally for the Brazilian population. Sociodemographic, lifestyle and comorbidity information was also collected. Chronic diseases and indicators of musculoskeletal disorders (MSDs) were self-reported. The study population consisted primarily of individuals between 30 and 45 years of age (78%), of self-classified races black or brown (96.2%), with no more than an elementary school education (77%) and married (64.6%). In all the SF-36v01 dimensions, the values in the sample were lower than in the general population of Brazil, which was used as the reference population. In the “Physical Health” domain (Physical Functioning; Physical Role Limitations; Bodily Pain; General Health Perception) a tendency toward a lower health-related quality of life was observed among those who were older, had a lower education level, and had a prevalence of MSDs, hypertension or arthritis. The interference of health conditions linked to the fisherwomen’s work activities may contribute to lower HRQOL in all analyzed aspects, in comparison to the general population. In light of these findings, public health policies must consider these informal workers who contribute greatly to Brazil’s economy and food system.

## 1. Introduction

Analyzing the quality of life of artisanal fisherwomen is extremely important in light of the knowledge that there are a range of environmental and work-related conditions that impose serious health risks [1]. Also quality of life correlates with labor demands, especially among informal workers that are at the margins of Brazil’s public health and social security policies. Quality of Life is a complex conceptual term that reflects individual and community knowledge, experiences and values [2,3]. The term is widely applied to many fields [4] and has a long history of use in many regions of the world [5]. According to the World Health Organization (WHO), quality of life is defined as “the individual’s perception of their position in life within the value system and cultural context in which they live, and in relation to their goals, expectations, standards and concerns” [6]. Among the advantages of measuring health-related quality of life (HRQOL) are the possibility to inform interventions and public health policies to systematically improve health among individuals and particular populations suffering from injury or disease [7].

A universally accepted tool to measure HRQOL is the Medical Outcomes Study 36-Item Short-Form Healthy Survey (SF-36v01), a generic instrument that has already been transculturally adapted for the Brazilian population. The survey is composed of 36 questions subdivided into eight sections: physical functioning, physical role limitations, bodily pain, general health perceptions, vitality, social functioning, emotional role limitations and mental health [8]. Overall, HRQOL incorporates pathology indicators, functional states of health (including physical, psychological and social functions) and the perception of health [2]. In Brazil, based on the registrants of the online survey portal, few Brazilians are using the SF-36v01 instrument to measure HRQOL. Furthermore, there are limited studies on the HRQOL of artisanal fishermen.

Artisanal fishing is a traditional activity benefiting from the extensive coastline and Brazilian fishing biodiversity. For every 200 Brazilians, one is an artisanal fisherman. According to estimates from the Ministry of Fisheries, Brazil has almost one million artisanal fishermen [9]. The northeastern region has the greatest number of fishermen in Brazil, with 372,787 registered, representing 43.7% of the country’s total [10,11] and providing approximately 75% of the fish in the region [11]. The state of Bahia, particularly the Todos os Santos Bay subregion, is home to a significant number of these fishermen [1]. In 2011, data from the Brazilian Institute of Geography and Statistics (IBGE) [12] estimated that, of the municipalities belonging to Todos os Santos Bay, approximately 11,850 individuals practice fishing as a main productive activity, which is approximately 33.8% of the total of registered fishermen in the state. Saubara, the municipality represented in this study, has the second largest percentage of people in the region dedicated to fishing, the majority of whom are of women [13].

Artisanal fishermen are generally members of traditional communities. They are culturally distinct groups that are recognized as such, having their own forms of social organization, occupying and using the land and natural resources in their cultural, social, religious, ancestral and economic reproduction, and using knowledge, innovation and practices generated and transmitted through tradition [1]. As informal workers, they have autonomy to make decisions about their work, and are responsible for providing their own tools and for all states of production. They usual harvest enough fish or seafood for subsistence and the remaining is used for income generation, often selling to an intermediary that takes the products to market. Association membership constitutes a major benefit among this low-income population because they gain access to special benefits that the federal government grants only to registered fishermen. These benefits include retirement benefits based on age and special income during fishery conservation periods to encourage fishermen to reduce fishing activity. They also may access financial aid through a social welfare for low-income Brazilian families [14].

Fishing is an occupation that involves ergonomic, physical, chemical and biological risks [1]. Generally, fishermen work very long hours and continue to work even in the face of bodily pain, ailments (including musculoskeletal disorders, MSDs) and illness. Despite the significant proportion of the population comprised by artisanal fishermen and the health risks involved in their work activities that are known to compromise the quality of life, there are still few studies on this labor group in Brazil [15]. Furthermore, numerous international studies have assessed the importance of measuring HRQOL with environmental aspects and chronic diseases; however, few of them were conducted among fishery workers. Thus, the goal of this study is to assess the HRQOL of artisanal fisherwomen/shellfish gatherers from Saubara, Bahia in comparison to the general population.

## 2. Materials and Methods

### 2.1. Sample and Area

This is an epidemiological, cross-sectional cohort study [16] involving a sample of the population of artisanal fisherwomen/shellfish gatherers in the municipality of Saubara, Bahia. According to the 2010 census, Saubara has 11,201 residents [10], of which 48.9% are men and 51.1% are women. The economically active population (EAP) is 5196 people, 11% of which are artisanal fishermen registered with Saubara Association of Shellfish Gatherers. The total number of those registered between April and May of 2013 was 568 individuals, of which 426 (75%) were women and 142 (25%) were men. Our team previously determined that the great majority of artisanal fisherwomen/shellfish gatherers were registered in associations [17]. Therefore, 426 artisanal fisherwomen/shellfish gatherers approximate the total number of women workers in that sector in the municipality, and represents close to 7.4% of the female residents in Saubara, based on the population data from the 2010 census.

To calculate the sample size, a 50% prevalence and 5% error was used on the population total (*N*) of 426 artisanal fisherwomen/shellfish gatherers resulting in a minimum sample of 203 individuals. The final sample of 209 participants was obtained, accounting for 3% possible loss in samples. The sampling was random, simple and without replacement, where each individual was drawn using a random number chart. The following inclusion criteria were defined for the research participants: female, 18 years of age or older and at least one year of practicing the activity. Fisherwomen/shellfish gatherers who were selected but not actively shellfish gathering at the time were still given the opportunity to participate in case their absence from work was a result of injuries that they sustained from work. Their inclusion in the study was our effort to minimize the survival effect of healthy workers [16].

### 2.2. Measures

Data was collected between 10 April 2013 and 10 May 2013 by eight undergraduate students and four master’s students previously trained in ethical, theoretical and methodological research conduct. The selected artisanal fisherwomen were invited by their association to fill out a questionnaire about health, and were also informed about the broader research objectives that involved the pre-diagnosis of musculoskeletal disorders. Afterwards, all were referred to the Occupational Health Services clinic (SESAO) at the Professor Edgard Santos University Hospital (HUPES) for specialized exams. The fisherwomen that did not participate in the study but had some kind of musculoskeletal complaint were also referred to the clinic. A structured questionnaire that was previously validated [18] was adapted for our studies with artisanal fisherwomen [17]. The questionnaire included the following items: identification; sociodemographic information; work information; current and previous occupational history; daily hours of work with shellfish; health-related habits like smoking, alcohol consumption, medication use and physical activity; co-morbidities; type and duration of housework; musculoskeletal symptoms; and physical and psychosocial demands at work.

The variables age, race, gender, education, and presence of diabetes, hypertension or arthritis were self-reported and collected as such. The overall musculoskeletal disorders (MSDs) variable was selected in accordance with previous studies carried out with this population [17,19] in which the overall MSDs was defined as a record of pain or discomfort in various anatomical regions in the last twelve months, with a minimum duration of one week or a minimum monthly frequency, not caused by acute injury. Symptoms had to be described using at least one of the following: degree of severity ≥3 on a scale of 0 to 5 (0 indicating no discomfort and 5 indicating unbearable pain); seeking medical attention because of the problem; absence from work (official or otherwise); change of work for health restrictions [20,21].

The SF-36v01 questionnaire is composed of 36 questions divided into eight domains (physical functioning; physical role limitations; bodily pain; general health perception; vitality; social functioning; emotional role limitations; mental health) [8]. For the current study, priority was given to using the first four domains to specifically address physical health components. The selection of those domains was the result of the need to assess and compare obtained information with that of already developed research that focused on that population. The versatility of its application for people over the age of 14 years, with reliability and validity levels exceeding the recommended minimum standards, makes this instrument appealing to use in combination with other questionnaires [22]. Table 1 presents the items analyzed from the SF-36v01 questionnaire that correspond to the two measurement groups assessed: physical health and mental health.

### 2.3. Statistical Analyses

With the data collected, selected domains were analyzed with the support of software provided by the organization that created the instrument, concomitantly using the statistical package R version 3.1.3 (Free Software Foundation, Boston, MA, USA).

Our analysis began with a descriptive analysis of each domain, as well as the calculation of specific scores ranging from 0 to 100, values representing the lowest and highest quality of life, respectively.

Afterwards, the simple frequencies, central tendency (mean, median) and dispersion (standard deviation) were calculated for each domain. The selection criteria for categorical variables compared with the scores of the domains found were similar to those used in other HRQOL studies undertaken among the Brazilian population, and in international studies involving artisanal fishermen [7,22,23,24].

In order to compare two averages, a simple *t*-test was used when the assumption of normality was satisfied; otherwise, the Wilcoxon test was used. To compare three or more averages, we used analysis of variance (ANOVA) followed by the Tukey test, when the statistical assumptions were met and the null hypothesis was rejected; otherwise, the Kruskal-Wallis test was used and, when necessary, a nonparametric multiple comparison test. Statistical significance was classified as *p*-value less than or equal to 0.05.

### 2.4. Ethical Aspects

This research was submitted and approved by the Committee on Research Ethics in Human Beings (CEP) at the Faculdade de Medicina da Bahia (No. 356.261). After the authorization from CEP/Universidade Federal da Bahia (UFBA), a request to use the SF-36v01 instrument was submitted to the organization that created the questionnaire, obtaining license No. QM02558898. All research participants signed informed consent documents.

## 3. Results

A total of 209 artisanal fisherwomen/shellfish gatherers participated in this study with no refusals. The average age of participants in the study was 39.6 years of age (SD ± 11.5). Table 2 shows the sociodemographic characteristics and the health conditions of the population randomly selected for the sample. The study population is comprised primarily of women between the ages of 30 and 44 (or older), representing 78% of interviewed shellfish gatherers; moreover, a majority self-reported being of black or brown race (96.2%), are predominantly married (64.6%) and have no more than an elementary school education (77%). Among the chronic diseases mentioned, there was an overall MSDs prevalence (in all regions of the body) of 94.7% (198), followed by 27.3% (57) hypertension, 10.5% (22) arthritis, and 6.7% (14) diabetes.

Table 3 shows a descriptive statistical analysis of the domains from the SF-36v01 questionnaire including the central tendency and dispersion measurements, as well as the asymmetry, ceiling and floor effects for each domain. The mean obtained for each domain was below 66.44. The asymmetry of data (skewness) from six domains was negative, showing that the majority of shellfish gatherers surveyed had below-average values for the domains.

**Figure 1:**
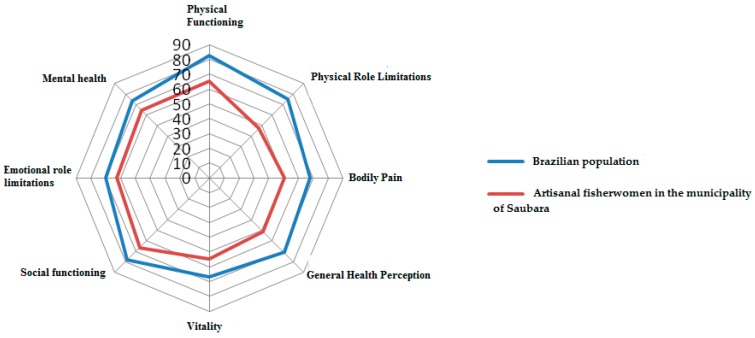
SF-36v01 scores from artisanal fisherwomen/shellfish gatherers from Saubara, Bahia, Brazil in relation to the Brazilian population. Source: Authors’ calculations.

Figure 1 represents a comparison of domain values from SF-36v01 of the artisanal fisherwomen/shellfish gatherers in relation to normative reference values of the Brazilian population highlighted in other scientific studies. According to the polar graph representation, it is clear that these workers from Saubara had, in all areas, lower scores when compared to the results of the Brazilian population, according to Laguardia *et al*. [24].

Table 4 shows the SF-36v01 domains with respect to age, education and chronic diseases (self-reported). Within the abovementioned variables, statistically significant (*p* ≤ 0.05) differences in SF-36v01 scores were observed for many of the domains. One can perceive the tendency of the mean SF-36v01 scores in all domains to decrease as age increases, distinguishing the 45 years or older group according to statistical tests (multiple comparisons). In the schooling variable, the increase of years in school (nine years or more) had, in general, the highest mean of the domain scores in SF-36v01, while only the physical functioning domain was statistically significant (*p* ≤ 0.000).

The scores for physical functioning, physical role limitations, bodily pain and social functioning domains were statistically significant between women who were identified as having overall MSDs versus those who did not. The prevalence of MSDs in this population was 94.7%. This variable was the only one among chronic diseases that used research from validated instruments, according to previous studies from our group [17,19]. The presence of diabetes presented low scores; however, most statistical tests demonstrated no statistical significance. It is noteworthy that physical functioning, physical role limitations, bodily pain and general health perceptions were the only statistically significant domains in relation to hypertension, as reported in Table 4. All of the domains except for mental health showed statistically significantly lower scores among participants with arthritis compared to participants without.

## 4. Discussion

This is the first study to assess the health-related quality of life (HRQOL) among artisanal fisherwomen in Saubara, Bahia. Our findings show that self-reported HRQOL is significantly lower than that of the general population. Furthermore, the SF-36v01 scores of the artisanal fisherwomen suggest that the worst health status is among the population that is 45 years of age or older, with four or less years of schooling and chronic diseases, especially MSDs, hypertension or arthritis.

These findings confirm the results of previous studies carried out in the Brazilian population [22,24], whereby individuals (mainly women) with a lower degree of education and self-reported chronic medical conditions presented a greatly compromised health status. There is evidence that health status is associated with quality of life and that the characteristics of life in society, including social, psychological and environmental contexts, are related to one’s level of health [25].

In comparing the findings of this study to that of a similar implementation of SF-36v01 in the general population, we found that artisanal fisherwomen presented lower scores in all eight domains of the survey [22]. In the description of data for the Brazilian population, the mean scores range from 66.85 (vitality) to 82.45 (physical functioning), while the results found in this study range from 47.3 (physical role limitations) to 66.4 (physical functioning). These findings are supported by other studies conducted amongst fishermen in comparison to the larger population. For example, the shellfish gatherers of Galicia showed a lower score than the reference population of Spanish women in five of the eight domains of the SF-36 instrument [7]. Similarly, in Brazil, the artisanal fisherwomen from the Mosqueiro community in the city of Aracaju, had lower scores in the physical functioning domain than the non-fishers of that community [26]. This suggests that there may be aspects intrinsic to the work of artisanal fishing that can deteriorate one’s health. It is important to note that nationally published quantitative (indexed) studies that analyze the HRQOL of artisanal fishers were not found. According to the literature review conducted by Rios *et al.* [15], there are few epidemiological studies in the literature that describe these informal workers and the relationships between health and the craftsmanship processes in traditional and self-employed categories.

In relation to age, this study demonstrates that older artisanal fisherwomen report the worst HRQOL in all domains, with the most unfavorable values being reported by those 45 years and older range. Interestingly, in comparison to the national reference, HRQOL reporting by age group diverges, with individuals of ages 30 to 44 having the worst health [22,24]. It is important to highlight that the artisanal fisherwomen/shellfish gatherers are exposed early in life to long workdays, beginning in childhood and are exposed to repetitive movements, excessive force, awkward postures and long hours in standing or sitting positions [1]. Thus, the accumulation of physical and mental stresses over the life-course from their occupation may be evident by the lower HRQOL among the older fisherwomen.

With the exception of diabetes, the presence of chronic diseases and pathological conditions seemed to be directly associated with HRQOL of shellfish gatherers, especially in the physical health block. Scores for physical functioning, physical role limitations, bodily pain domains and general health perception were all low among fisherwomen that had overall MSDs, hypertension, and arthritis. This confirms results from a previous study that states that the presence of chronic diseases, particularly MSDs, has a major impact on the quality of life [27]. There is evidence that the occupation of shellfish gathering has several intrinsic hazards and evidence for, as well as a causal relationship between physical exertion at work and MSDs related to work [20,21]. In our previous studies conducted with this population, the association between exposure to the work of shellfish gathering and the occurrence of MSDs was revealed [17,19]. From Roux *et al.* [27], the presence of MSDs and its initial impact deteriorates quality of life, making the affected individual unfit to carry out the duties of daily life, as bodily pain received the lowest scores among all domains on the SF-36 instrument. In our study, the lowest scoring domains were physical role limitations and bodily pain in the presence of overall MSDs.

According to Yeng *et al.* [28], there are varying degrees of predisposition to chronic pain syndromes related to an individual’s cardiovascular and musculoskeletal condition, physical constitution, sexual characteristics, psychological behavior profile, degree of stress and satisfaction with the workplace, family and society, the reinforcement of a disabled condition and personal perceptions of wellness. According to Pena *et al.* [1], artisanal fisherwomen/shellfish gatherers are exposed to risks during childhood, as they wield work instruments, are exposed to UV radiation from the sun and weather-related hazards and work within rocky and/or mangrove areas without protection. Risks related to external factors accumulate over time and add to the conditions that negatively impact their life.

Another important finding of our study was that the artisanal fisherwomen reported on average much lower scores for physical domains of health than for mental domains. These findings corroborate previous studies in fisherwomen where physical health was clearly the most affected [7]. Though the mental health scores of artisanal fisherwomen are the highest scores of the entire applied instrument, they are still lower than those of the Brazilian population. This could be related to the physical burden of this work as well as the accumulation of housework, thus making the daily lives of these women exhausting and highly dependent on their overall state of health.

### 4.1. Strengths and Limitations

There are several strengths of this study. The analysis of the SF-36v01 data applied to this population suggested good performance for the applicability of this instrument, mainly to assess the perception of health and health-related quality of life. Another strength of this study is the fact that we studied HRQOL in the general population, which is rare. Many of the published studies assess HRQOL in patients with a particular disease or health disorder. Some of the limitations of this study are that HRQOL is self-reported by the individual and might be influenced by psychological phenomena, such as adaptation to the illness, leading to challenges in interpreting HRQOL measures, especially in cross-sectional studies. A limitation to this type of epidemiological study design can be overcome by conducting prospective studies in this population instead.

### 4.2. Recommendations

In general, artisanal fishermen, similar to other sectors of informal work, are at the margins of public health policies, as they are often not adequately protected from occupational risks, and there are no policies within the Brazilian National Health System (SUS) that guarantee protections similar to those established for formal workers [14]. The results of this study, which show a low quality of life among artisanal fisherwomen compared to the population in general, should be used for the implementation of compensatory public health policies, training of health professionals and technicians within SUS, and evaluating the allocations of federal retirement benefits. Also, because this group of informal workers comprises a significant percentage of the workers in northeastern Brazil, much more research needs to be conducted to understand the range of occupational-related exposures and injuries among small-scale fishermen.

## 5. Conclusions

This study assessed the health-related quality of life (HRQOL) of artisanal fisherwomen/shellfish gatherers from Saubara, Bahia. The vulnerability of this group of workers with regard to health maintenance—expressed by the term health-related quality of life—was revealed quantitatively. From this perspective, analysis of HRQOL with the use of quantitative methods allowed us to outline a sociodemographic and health profile of this group of workers and make comparisons with the general population. The study revealed that HRQOL is considerably diminished in this group of workers, especially in the dimensions of physical health, which is potentially related to their exhausting workload. Physical functioning, physical role limitations, bodily pain and general health perception components were reported as low, thus contributing to an overall low HRQOL when compared to the Brazilian population at large. The artisanal fisherwomen with the least amount of schooling, oldest age and chronic medical conditions reported the lowest HRQOL scores of the participants in the study. In light of these findings, public health policies and social benefits must be more inclusive of these informal workers, who contribute greatly to local economies and food systems in Brazil.

## Figures and Tables

**Table 1:** Description of domains from the SF-36v01 questionnaire.

Domain	Questionnaire Items	Measures Evaluated	Measures
Physical Functioning	3a, 3b, 3c, 3d, 3e, 3f, 3g, 3h, 3i, 3j	Assess the presence and extent of the restrictions imposed on physical capacity	Physical health
Physical Role Limitations	4a, 4b, 4c, 4d	Assess physical aspects	
Bodily Pain	7, 8	Based on a question from the SF-20 questionnaire regarding pain intensity plus the interference of pain in daily activities	
General Health Perceptions	1, 11a, 11b, 11c, 11d	Derived from the General Health Rating Index survey	
Vitality	9a, 9e, 9g, 9l	Consider energy level and fatigue, derived from the Mental Health Inventory (MHI) questionnaire	Mental health
Social Functioning	6, 10	Assess the integration of the individual in social activities	
Emotional Role Limitations	5a, 5b, 5c	Assess emotional wellbeing	
Mental health	9e, 9d, 9f, 9h	Investigate the dimensions of anxiety, depression, changes in behavior or emotional imbalance and psychological wellbeing; Summary of 38 items from the Mental Health Assessment Questionnaire (MHI-38)	

Source: Author adaptation, available questionnaire from SF-36 [23].

**Table 2:** Sociodemographic characteristics and the health conditions of artisanal fisherwomen/shellfish gatherers from the municipality of Saubara-Bahia, Brazil, 2013 (*n* = 209).

Sociodemographic Characteristics	*n* (209)	%
**Age**		
20–29	47	22
30–44	94	45
45–64	68	33
**Race**		
Black	125	59.8
Brown	76	36.4
White	8	3.8
**Civil Status**		
Single	56	26.8
Married/Unmarried Couple/Living Together	135	64.6
Separated/Widow	18	8.6
**Schooling**		
Up to 4 years	48	23
From 5 to 8 years	94	45
From 9 to 11 years	67	32
**Health conditions**		
*Overall musculoskeletal disorders*		
Yes	198	94.7
No	11	5.3
*Diabetes*		
Yes	14	6.7
No	195	93.3
*Hypertension*		
Yes	57	27.3
No	152	72.7
*Arthritis*		
Yes	22	10.5
No	187	89.5

Source: Authors’ calculations.

**Table 3:** Descriptive statistics of the SF-36v01 domains of artisanal fisherwomen/shellfish gatherers from the municipality of Saubara, Bahia, Brazil, 2013.

Descriptive Statistics	PF ^1^	PRL ^2^	BP ^3^	GHP ^4^	Vitality ^5^	SF ^6^	ERL ^7^	MH ^8^
Mean	65.7	47.3	50.44	51.2	54.5	66.4	62.4	64.8
Standard Deviation	24.9	40.8	25.3	21.8	22.9	30.5	42.9	23.5
Median	7.0	50.0	51.0	34.0	55.0	71.0	100.0	68.0
*Skewness* ^a^	−0.531	0.091	0.427	−0.131	−0.054	−0.566	−0.508	−0.51
*Floor* ^b^	0.96	33.0	2.4	1.4	0.5	4.8	26.3	0.96
*Ceiling* ^c^	8.1	26.8	12.0	1.4	0.96	27.8	50.2	7.7

Source: Authors’ Calculations. Notes: ^1^ Physical Functioning; ^2^ Physical Role Limitations; ^3^ Bodily Pain; ^4^ General Health Perception; ^5^ Vitality; ^6^ Social Functioning; ^7^ Emotional Role Limitations; ^8^ Mental health. ^a^ Asymmetry; ^b^ Floor effect; ^c^ Ceiling Effect.

**Table 4:** Mean and standard deviation of the SF-36v01 instrument scores for sociodemographic categorical variables and chronic diseases with *p*-value of parametric and non-parametric tests of artisanal fisherwomen/shellfish gatherers in the city of Saubara, Bahia, Brazil, 2013.

**Variables**	**Physical Functioning (PF)**			**Physical Role Limitations (PRL)**			**Bodily Pain (BP)**			**General Health Perception (GHP)**		
	**Mean**	**SD**	*p*-Value *	**Mean**	**SD**	*p*-Value *	**Mean**	**SD**	*p*-Value *	**Mean**	**SD**	*p*-Value *
**Age** ^1,7^												
20 to 29 years ^a^	79.8	17.4		60.1	39.9		65.6	25.0		59.2	19.9	
30 to 44 years ^a^	70.1	21.6	0.000	51.6	40.8	0.000	47.2	23.0	0.000	52.3	21.6	0.001
45 or older ^b^	49.7	25.6		32.4	37.4		44.4	24.7		44.3	21.4	
**Schooling** ^1,7^												
Up to 4 years ^b^	51.04	26.8		33.9	37.4		42.9	24.3		47.3	21.4	
5 to 8 years ^a^	67.8	23.7	0.000	48.4	42.6	0.028	49.8	25.7	0.251	47.3	21.4	0.248
9 years or more ^a^	73.06	21.0		55.2	38.8		56.7	24.2		54.1	22.0	
**Overall MSDs** ^2^												
No	84.6	15.7	0.006	81.8	31.8	0.004	79.9	26.1	0.000	63.0	16.9	0.067
Yes	64.6	25.0		45.3	40.5		48.8	24.3		50.6	21.9	
**Diabetes** ^3,6^												
No	65.8	25.2	0.270	47.1	40.8	0.960	50.8	25.8	0.657	51.4	21.8	0.604
Yes	58.2	26.6		48.2	43.3		45.4	18.4		48.5	21.8	
**Hypertension** ^4,6^												
No	68.1	24.6	0.000	51.9	40.4	0.006	53.5	25.7	0.005	54.8	21	0.000
Yes	55.6	25.8		34.7	39.7		42.3	22.5		41.7	21	
**Arthritis** ^5,6^												
No	67.7	24.8	0.000	49.2	41.2	0.024	51.9	25.1	0.012	52.3	21.5	0.060
Yes	45.7	22.3		30.7	33.6		37.6	23.9		42.6	22.4	
												
**Variables**	**Vitality**			**Social Functioning (SF)**			**Emotional Role Limitations (ERL)**			**Mental Health (MH)**		
	**Mean**	**SD**	*p*-Value *	**Mean**	**SD**	*p*-Value *	**Mean**	**SD**	*p*-Value *	**Mean**	**SD**	*p*-Value *
**Age** ^1,7^												
20 to 29 years ^a^	56.8	22.9		72.7	24.7		74.5	39.0		65.5	20.6	
30 to 44 years ^a^	55.6	21.8	0.470	68.2	30.0	0.126	64.5	42.0	0.012	67.4	23.5	0.193
45 or older ^b^	51.5	24.3		59.7	33.7		50.1	44.0		60.8	25.0	
**Schooling** ^1,7^												
Up to 4 years ^b^	47.3	21.4		59.3	34.4		63.2	42.0		59.5	25.0	
5 to 8 years ^a^	51.1	21.7	0.138	69.8	30.6	0.188	55.7	44.0	0.072	66.7	24.0	0.223
9 years or more ^a^	54.2	22.0		66.9	26.7		71.1	41.0		66.2	21.0	
**Overall MSDs** ^2^												
No	64.6	19.3	0.133	87.0	19.6	0.016	81.8	41.0	0.081	81.8	40.5	0.081
Yes	53.9	22.9		65.3	30.6		61.3	43.0		64.2	23.8	
**Diabetes** ^3,6^												
No	54.5	22.9	0.916	66.5	30.5	0.957	62.9	43.0	0.490	64.6	23.5	0.685
Yes	54.3	22.3		66.4	31.9		54.8	46.0		66.9	23.6	
**Hypertension** ^4,6^												
No	55.9	22.8	0.152	68.6	28.7	0.180	65.8	42.0	0.061	66.9	23.0	0.025
Yes	50.8	22.8		60.7	34.5		53.2	44.0		59.0	23.7	
**Arthritis** ^5,6^												
No	55.7	22.6	0.030	68.9	29.3	0.001	65.4	42.0	0.003	65.0	23.5	0.635
Yes	44.6	23.2		44.9	32.2		36.3	41.0		62.9	23.7	
												

Source: Authors’ calculations. Notes: ¹ Kruskal Wallis Test (PF, PRL, BP, Vitality, SF, ERL and MH) and ANOVA (GHP); ^2,3,5^ Wilcoxon Test (PF, PRL, BP, GHP, Vitality, SF, ERL and MH); ^4^ Wilcoxon Test (PF, PRL, BP, Vitality, SF, ERL and MH) and *t*-test (GHP); Standard Deviation (SD); * 5% Significance; ^6^ Self-reported; ^7^ Multiple comparisons test a = a; b ≠ a.
